# Health Means Dollars

**Published:** 1883-07

**Authors:** 


### HEALTH MEANS DOLLARS.

IT is cash in one's pocket to study the care of the body. Is there any better gift in this world than a sound, healthy body? Is there any enjoyment in the world's good things without one? Wouldn't many a rich man give half his wealth could he but exchange his worn, wan, wasted body for that of the man who shovels in his coal, who has a vigorous appetite for everything set before him and who never after eating thinks of his stomach? If new, fresh, strong bodies could be bought as easily as new suits of clothes, wouldn't there be a run on the wholesale and retail body stores? Yet nature can do a great deal in the way of repairing bodies and keeping them sound, providing her hints are taken and her demands heeded. Every pain, every ache, every fatigue, every premonitory weakness or failing of organ or function is such a demand. Now she calls for rest, then for some new element or condition to repair waste. Is it often granted? Nature gives one order, business and necessity exact quite another. Nature says: "Keep to-day out of the store, the shop, the lawyer's office, the editorial room. Get into you some sunshine, some fresher air and a change of conditions and surroundings." Business says: "No. Work in the same old way. Breathe the same semi-putrid air. Exercise the same old jaded faculty or department of brain, and when the machine at last breaks down and you can't crawl, send for the doctor." If one-third the attention that is readily granted to making money were bestowed on the care of the body, as regards the nourishment which will give it more strength, the amount and kind of labor best suited for it, and the condition and location most suited for it, there would be better work both mental and physical, vigor would be prolonged, men and women would not be useless at sixty, and the world would behold a race superior in all respects to any existing in the known past. But ere this is realized our "wise men" must learn to live a great deal more outside of their closets, and our business men outside of their counting rooms and exchanges. Any occupation that puts on a man's face the color of tallow or parchment is a health-destroying business.

---

DR. HAWKS, an eloquent and popular New York divine, once asked the vestrymen of his church to increase his salary because of his increased family expenses. "Don't trouble yourself," said the vestryman, "the Lord has said he will care for the young ravens when they cry." "I know that," said the clergyman, "but nothing is said about the young Hawks."

